# Microstructural alterations in medial forebrain bundle are associated with interindividual pain sensitivity

**DOI:** 10.1002/hbm.25281

**Published:** 2020-11-10

**Authors:** Maria Geisler, Elizabeth Rizzoni, Nikolaos Makris, Ofer Pasternak, Yogesh Rathi, Sylvain Bouix, Marco Herbsleb, Karl‐Jürgen Bär, Thomas Weiss, Zora Kikinis

**Affiliations:** ^1^ Department of Clinical Psychology Friedrich‐Schiller‐University Jena Jena Germany; ^2^ Psychiatry Neuroimaging Laboratory, Department of Psychiatry Brigham and Women's Hospital, and Harvard Medical School Boston Massachusetts USA; ^3^ Departments of Radiology and Psychiatry Harvard Medical School Boston Massachusetts USA; ^4^ Departments of Radiology and Psychiatry Massachusetts General Hospital Charlestown Massachusetts USA; ^5^ Department of Sports Medicine and Health Promotion Friedrich‐Schiller‐University Jena Jena Germany; ^6^ Department of Psychosomatic Medicine University Hospital Jena Jena Germany

**Keywords:** diffusion magnetic resonance imaging (dMRI), endogenous pain modulation, fractional anisotropy (FA)

## Abstract

The perception of pain to noxious stimuli, also known as pain sensitivity, varies among individuals. The comprised brain structures and their white matter pathways are complex and elusive. Here, we aimed to investigate whether variation of microstructure of the medial forebrain bundle (MFB), a tract connecting the basal forebrain with the brain stem, is associated with interindividual pain sensitivity. We assessed interindividual pain sensitivity as a rating of pain intensity to heat stimuli (45, 47, and 48.9°C) in 38 healthy men (age: 27.05 ± 5.7 years). We also reconstructed the MFB using multitensor tractography from diffusion magnetic resonance imaging (dMRI) and calculated free‐water corrected dMRI measures of fractional anisotropy (FA_t_), radial diffusivity (RD_t_), and axial diffusivity (AD_t_). Lower ratings of interindividual pain intensity correlated with higher FA_t_ and lower RD_t_ of the MFB. As changes in FA_t_ and RD_t_ may reflect abnormalities in myelination, the results might be interpreted as that a lower pain rating is associated with higher degree of myelination of the MFB and could represent an inhibitory pathway of pain. Our results suggest that alteration of microstructure in the MFB contributes to the interindividual variation of pain perception.

## Graphical abstract

We aimed to investigate whether variation of microstructure of the medial forebrain bundle (MFB) is associated with interindividual pain sensitivity, by assessing interindividual pain sensitivity as a rating of pain intensity to heat stimuli and reconstructing the MFB using multitensor tractography from diffusion magnetic resonance imaging (dMRI) in 38 healthy men. Lower ratings of interindividual pain intensity correlated with higher fractional anisotropy (FA_t_) and lower radial diffusivity (RD_t_) of the MFB. As changes in FAt and RDt may reflect abnormalities in myelination, the results might be interpreted as that a lower pain rating is associated with higher degree of myelination of the MFB and could represent an inhibitory pathway of pain.

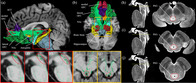

## 1 INTRODUCTION

The neuromatrix of pain (Iannetti & Mouraux, 2010) is a complex network comprised of ascending and descending pathways. The ascending pathways transmit nociceptive information from the periphery to the brain. This transmission can be modulated by descending pathways leading to an altered perception and experience of pain (Ossipov, Dussor, & Porreca, 2010; Tracey & Mantyh, 2007). The descending system consists of several areas in the limbic forebrain, including the medial, orbitofrontal, and dorsolateral prefrontal cortex (mPFC, OFC, DLPFC), the anterior cingulate cortex (ACC), nuclei in the amygdala, and the hypothalamus (Ossipov et al., 2010). These limbic forebrain structures project to the periaqueductal gray (PAG). PAG is the best known control region of the descending system and is located in the midbrain (Linnman, Moulton, Barmettler, Becerra, & Borsook, 2012). The PAG projects to the rostral ventromedial medulla, which further projects to dorsal horn neurons. The neurons of the descending pathway can inhibit or facilitate pain, such that the person experiences less or more discomfort (Heinricher, Tavares, Leith, & Lumb, 2009).

One way to assess the activity of the descending system is to measure pain sensitivity. Pain sensitivity describes the reaction to various standardized noxious stimuli (Ravn, Frederiksen, Skovsen, Christrup, & Werner, 2012) and has been shown to vary between subjects (Nielsen et al., 2008). There are several factors (e.g., ethnic (Campbell, Edwards, & Fillingim, 2005), physical (Abrishami, Chan, Chung, & Wong, 2011) psychological (Baum, Huber, Schneider, & Lautenbacher, 2011; Oosterman, Dijkerman, Kessels, & Scherder, 2010), genetic (Afari et al., 2011), and social (Vigil et al., 2013) that influence pain sensitivity. However, the exact neuronal mechanisms (e.g., white matter tracts of the descending system) that underlie the variability of pain sensitivity are only fragmentarily understood. A major fiber tract connecting lateral and medial OFC, DLPFC, ACC, amygdala, and hypothalamus with the ventral tegmental area (VTA) and brain stem (and vice versa) is the medial forebrain bundle (MFB) (Coenen et al., 2018; Coenen, Panksepp, Hurwitz, Urbach, & Madler, 2012). The MFB has been studied so far in affective disorders and addiction as this tract also mediates reward and motivation (Bracht, Linden, & Keedwell, 2015; Rivas‐Grajales et al., 2018; Russo & Nestler, 2013; Wise, 2005). However, the neural substrates of the descending pain system are the same as those of the MFB, suggesting that this fiber tract is a part of that system. Variability between subjects and within the axons of the MFB can potentially have an effect on the perception of pain. For instance, a higher degree of myelination or larger diameter of the MFB could exert higher modulation of endogenous pain. Consequently, the person would experience less pain.

The methodology of diffusion magnetic resonance imaging (dMRI) allows reconstruction of white matter tracts and evaluation of microstructural features of fiber tracts in vivo (Basser & Pierpaoli, 1996). The most common diffusion indices in dMRI are fractional anisotropy (FA), axial diffusivity (AD), and radial diffusivity (RD). A high value of FA (closer to 1) represents diffusion anisotropy (water molecules move faster in a certain direction) and, among other effects, may reflect fiber density and/or degree of myelination (Kingsley, 2006). To gain additional information about microstructural changes, AD and RD are used. In a series of animal experiments, it has been shown that FA decreases and RD increases in demyelinating axons (Song et al., 2003; Song et al., 2005), while FA and AD decreases with axonal degeneration (Song et al., 2003). Further, the specificity of these measures to tissue changes can be improved by eliminating free‐water (FW) contribution of the signal (Metzler‐Baddeley, O'Sullivan, Bells, Pasternak, & Jones, 2012; Pasternak, Sochen, Gur, Intrator, & Assaf, 2009).

The MFB has not been successfully delineated from dMRI until recently. While associations, projection and commissural fiber tracts could be reconstructed using the approach of single tensor tractography (Catani & Thiebaut de Schotten, 2008), white matter tracts connecting subcortical and cerebellar regions are much more difficult to reconstruct because of the presence of crossing and fanning fibers in this area. The problem can be solved by applying probabilistic or multitensor tractography (Malcolm, Shenton, & Rathi, 2010), approaches that were applied to reconstruct the MFB (Rivas‐Grajales et al., 2018; Zhang et al., 2020).

The relationship between brain white matter and pain sensitivity has been explored previously, but the findings are inconsistent. Several dMRI studies examined differences in FA in brains of patients with chronic pain and healthy controls, and report correlation of FA in specific white matter tracts with specific pain conditions (for review see (Martucci, Ng, & Mackey, 2014). Recently, Zhang et al. (2020) reconstructed nine brainstem fiber trajectories of pathways (including the medial forebrain tract [MFT]) that are potentially involved in pain modulation and reported no significant association between FA in the MFT and the assessed pain levels “pain right now,” and “worst pain in last month.” In contrast, the dMRI study examining the structural relationship of the white matter of the descending pain system and placebo hypoalgesia reported that pain sensitivity was associated with lower FA of white matter tracts connecting the PAG with the rostral ACC (rACC) and DLPFC in healthy subjects (Stein, Sprenger, Scholz, Wiech, & Bingel, 2012). The findings are promising and encourage investigation of the relationship between MFB and pain sensitivity. However, the later study only focused on FA as single outcome variable. Furthermore, the study has been restricted to explore the fiber connections of PAG with rACC and DLPFC, only.

In the present study, we aimed to investigate whether the microstructure of the MFB was associated with interindividual pain sensitivity as measured by pain intensity ratings to standardized physical stimuli. We explored the microstructure of the MFB in 38 healthy men using dMRI of the brain and applied the FW imaging method (Pasternak et al., 2009) to eliminate partial volume effects of FW CSF on dMRI measures. We expected that pain sensitivity of the individual will be inversely associated with FA_t_ in the MFB. Furthermore, to investigate whether myelination and/or increased number of tracts/larger diameter of the MFB might contribute to pain sensitivity, we examined the association of RD_t_ and AD_t_, in addition to FA_t_. We hypothesized that interindividual pain intensity rating will correlate negatively with FA_t_ and positively with RD_t_ if myelination of MFB plays a role, while interindividual pain intensity rating will correlate negatively with FA_t_ and negatively with AD_t_ if the number or size of axons in the MFB play a role. Additionally, we aimed to explore whether changes in FW in MFB were involved with pain.

## 2 MATERIALS AND METHODS

### 2.1 Participants

Participants were recruited by advertisements posted at the University of Jena, Germany. We only included male subjects in the study to prevent menstruation related influences to pain processing (Riley, Robinson, Wise, & Price, 1999). Inclusion criteria were as follows: age 18–40 years; BMI 18.5–30 kg/m^2^; no pain disorder, no psychiatric or neurological disease; MRI suitable. The final sample size included 38 healthy men (age: 27.05 ± 5.7 years). Subjects were paid for participation (25 €). The Ethics committee of the Faculty of Social and Behavioral Sciences of the Friedrich Schiller University Jena approved the study. All subjects signed informed consent.

### 2.2 Study design

**FIGURE 1:**
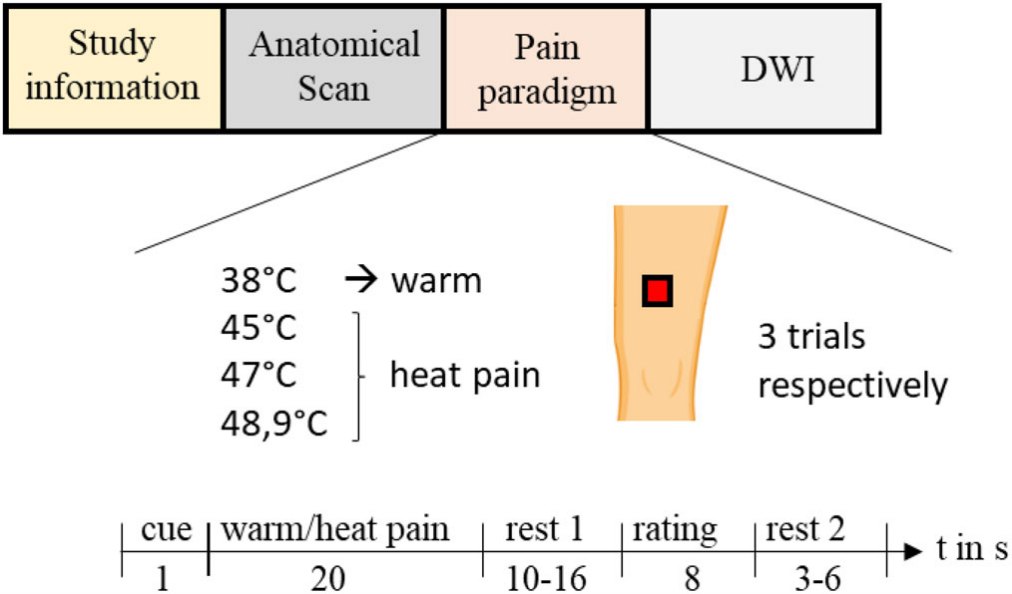
Study design. DWI, diffusion weighted images

After the subjects had been informed about the study's procedure, a high‐resolution anatomical scan of the whole brain was assessed. Then the pain paradigm was conducted followed by the acquisition of diffusion weighted images (DWI; see Figure 1).

#### 2.2.1 Paradigm to assess pain intensity

A pseudorandom sequence of twelve 20‐s thermal stimuli with different intensities (38, 45, 47, and 48.9°C; three trials each) was applied using a 27 mm diameter fMRI‐compatible Peltier thermode (PATHWAY Model, Contact Heat‐Evoked Potential Stimulator; Ramat Yishai, Israel). Each trial started with a visual cue “+.” After presenting the cue for 1 s, the thermal stimulus was administered for 20 s (approximately 1.5‐s ramp up, 17‐s plateau, approximately 1.5‐s ramp down) at the left thigh. After a short delay (10–16 s; Rest 1), participants had 8 s to rate the level of pain of each stimulus on the presented visual analogue scale (VAS) ranging from 0 = no pain to 100 = unbearable pain. A variable intertrial interval (3–6 s; Rest 2) followed before the start of the next thermal stimulation (see Figure 1).

### 2.3 MRI data acquisition and preprocessing

Anatomical and diffusion MRI sequences were performed with a 3 T MRI scanner (Siemens Magnetom Prisma fit, Erlangen, Germany) using a 64‐channel standard head coil. DWI were acquired using an echo planar image sequence with the following parameters: TR = 6,800 ms; TE = 57 ms; diffusion encoding directions = 81, *b* = 1,200 s/mm^2^; 72 slices, resolution = 1.7 × 1.7 × 1.7 mm. Additionally, a high‐resolution T1‐weighted anatomical scan (3D‐MP‐RAGE sequence, TE = 3.03 ms, TR = 2,300 ms, 192 slices, resolution = 1 × 1 × 1 mm) was used. An in‐house script was used to postprocess the MRI data of each participant in this study. The quality of the images was visually checked, and all images passed this test. Motion and eddy current correction of diffusion images was performed using an affine registration algorithm in FSL (http://www.fmrib.ox.ac.uk/fsl). Then structural and diffusion images were manually masked using 3DSlicer software, Version 5.2 (Surgical Planning Laboratory, Brigham and Women's Hospital, Boston, MA; http://www.slicer.org). The mask defines the area of the brain. For each DWI, we performed multitensor whole‐brain tractography with Free Water estimation (with the following parameter settings: minFA: 0.08, seedFALimit: 0.1, Qm (expected variance in orientation from one step to next): 0.001, Ql (expected variation in eigenvalues from one step to next): 50, Rs (expected noise level in data): 0.02, stepLength: 0.3, recordLength: 0.9, Qw (expected variance in FW fraction from one step to next): 0.0015, minGA (minimum generalized anisotropy): 0.08, seedsPerVoxel: 1) using the multifiber tracking method (Malcolm et al., 2010) (https://github.com/pnlbwh/ukftractography). The multifiber tracking method has very high reliability and reproducibility of tracing the same fiber connections across scans, across a wide age range, and across a variety of different acquisition protocols (Zhang et al., 2019). In addition, this method was able to reliably trace fibers through crossing fiber regions (Fillard et al., 2011) and is therefore suitable for the reconstruction of whole brain white matter, including tracts connecting subcortical and cortical structures. The output of the multifiber tracking was streamlines, with FA_t_, AD_t_, RD_t_, and the FW fraction at every point along the streamlines. The structural masks were then applied to generate a label map for white and gray matter parcellation using FreeSurfer software, Version 6.0 (Desikan et al., 2006; Fischl et al., 2002). This resulted in cortical and subcortical parcellations as described by Desikan et al. (2006) and Salat et al. (2009). After visual quality control, the FreeSurfer label maps were registered to the diffusion images using the advanced normalization tools (Avants, Epstein, Grossman, & Gee, 2008).

### 2.4 Extraction of the MFB

**FIGURE 2:**
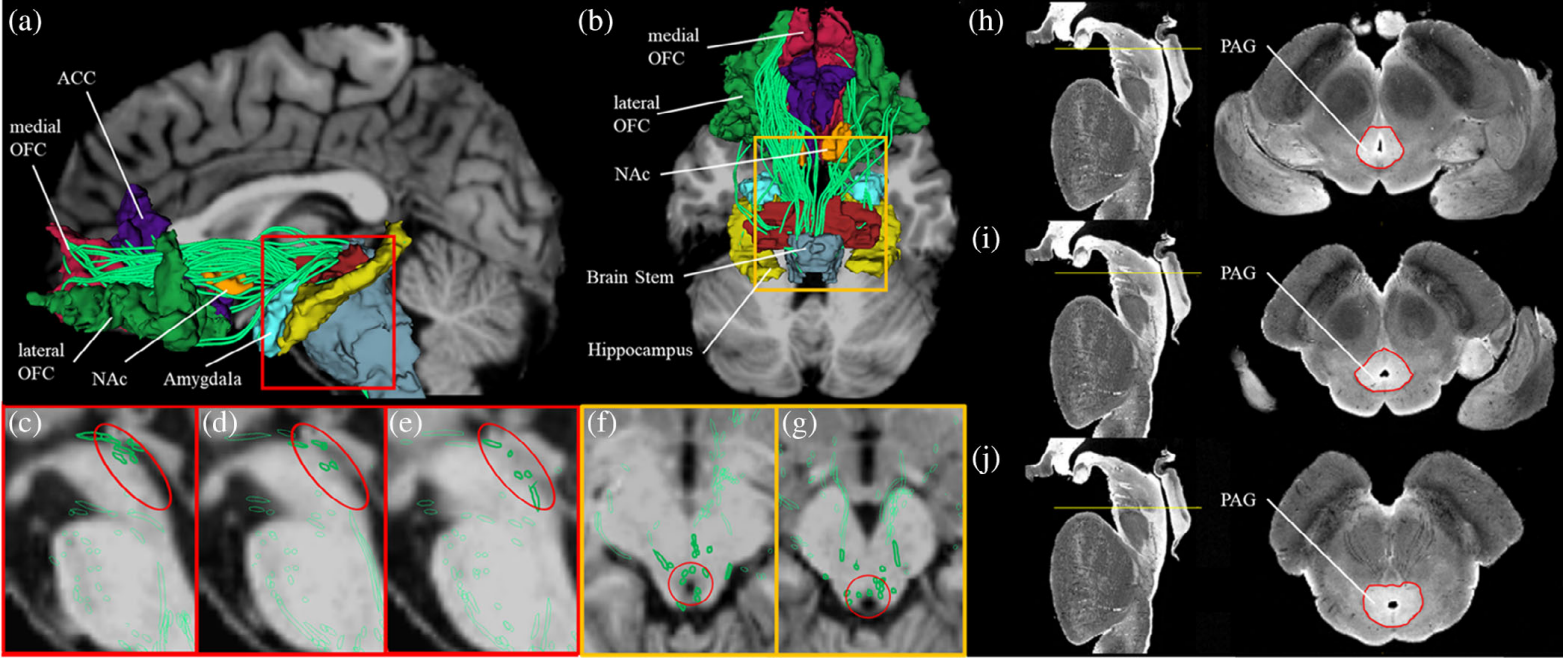
Medial forebrain bundle. Three‐dimensional reconstructions of the medial forebrain bundle (MFB). MFB fiber tracts are in green. In the background, structural MRI in sagittal or axial view are given in shades of gray. (a) Sagittal view of the MFB. (b) Axial view of the MFB. (c–e) Sagittal view of a subset of fibers of the MFB in close relationship with the anatomical location of periaqueductal gray (PAG) (circled in red). (f–g) Axial view of a subset of fibers of the MFB in close relationship with the anatomical location of PAG (circled in red). (h–j) Representation of PAG in a high‐resolution data set as transverse segmented sections through the brainstem. For each subfigure, a sagittal view of the brainstem is shown on the left with a yellow line representing the plane of the section that corresponds to the axial view on the right panel. PAG is segmented as shown in the paper by Makris et al. (2019) using the Calabrese et al. (2015) high‐resolution brainstem dataset. ACC, anterior cingulate cortex; NAc, nucleus accumbens, OFC, orbitofrontal cortex,

We delineated the MFB from DWI using the White Matter Query Language (WMQL). WMQL is a semi‐automated fiber delineation method that allows extraction of fiber tracts from whole‐brain tractography based on FreeSurfer labels (Wassermann et al., 2016). The MFB connects the PFC and the VTA. The VTA is split between two FreeSurfer labels, namely the brainstem and the ventral diencephalon (ventral DC). Including both labels (brainstem and ventral DC) as the seed region for WMQL of MFB would have been overinclusive. Therefore, we manually split the ventral DC in an anterior and a posterior part at the mammillary bodies, such that the bodies were not included in the posterior part. The posterior part of the ventral diencephalon and brain stem was the new VTA ROI, which also contains PAG, and we have visualized streamlines of the MFB in close relationship with the anatomical location of PAG in Figure 2. Similar to the study by Rivas‐Grajales et al. (2018), we seeded for MFB between the VTA (the posterior part of the ventral diencephalon and brain stem) and the following structures: nucleus accumbens (NAc), OFC (lateral and medial), ACC hippocampus, and amygdala (see Figure 2). We were able to track the projections in all 38 participants (number of streamlines: *M* = 51.4 ± 17.1, range: 32–129). To demonstrate specificity of the MFB in reducing pain, we have chosen the thalamooccipital tract as a control tract and tested the dMRI indices of this tract for associations to pain sensitivity. There were no statistically significant correlations between any of the dMRI indices of the thalamooccipital tract and pain intensity rating (see Table S1).

### 2.5 Statistical data analysis

All statistical analyses were performed using R version 3.4.1 (R Core Team, 2017). Significance levels were set to *p* ≤ .05. We defined the individual pain intensity rating as a mean of all nine‐pain ratings to applied heat pain stimuli (45, 47, and 48.9°C). We first tested whether there is a relationship between dMRI indices of the MFB and age by calculating Pearson correlations between age and FA_t_, RD_t_, AD_t_, and FW respectively. The correlations revealed no statistically significant relationships (FA_t_: *r* = −.06, *p* = .741; RD_t_: *r* = −.02, *p* = .902; AD_t_: *r* = −.18, *p* = .287; FW: *r* = .13, *p* = .439). Therefore, age was not included as covariate in subsequent correlations.

To test our hypothesis, we calculated Pearson correlations between brain measures of MFB (FA_t_, RD_t_, AD_t_, FW) and mean pain intensity rating, as data were normally distributed. Because multiple correlations were performed, we corrected the *p*‐values of FA_t_, RD_t_, AD_t_, and FW using the false discovery rate (FDR) procedure (Benjamini & Hochberg, 1995).

## 3 RESULTS

### 3.1 Descriptive data

On average, participants rated the pain intensity (0 = no pain, 100 = unbearable pain) of the applied noxious stimuli with 51.4 ± 17.1 (range: 15.6–87.2), indicating a large between‐subject variability in pain perception.

### 3.2 Correlation analyses of pain intensity and brain measures of MFB

**FIGURE 3:**
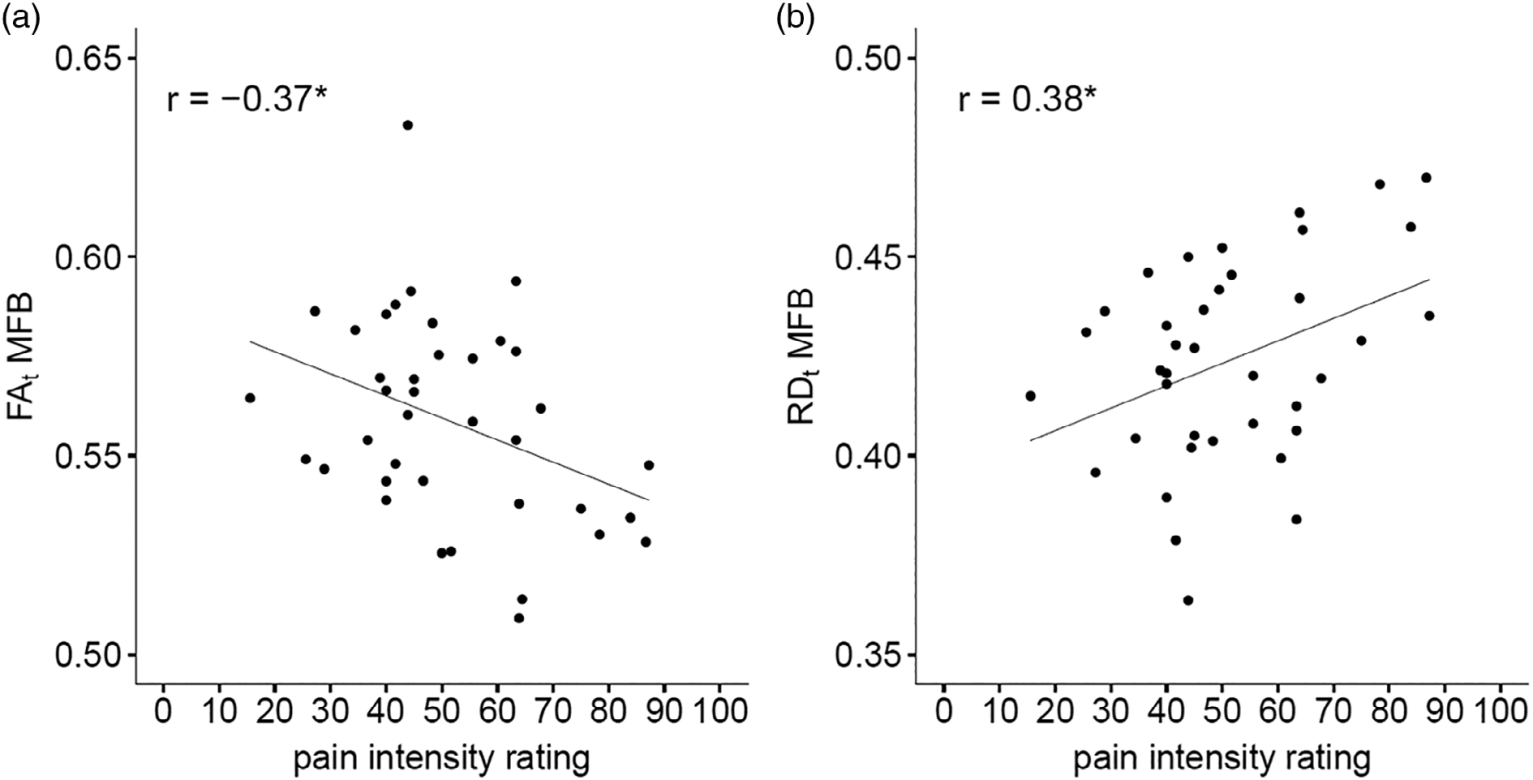
Association analysis. Correlation between pain sensitivity and free‐water corrected fractional anisotropy (FA_t_) (Panel a) and free‐water corrected radial diffusivity (RD_t_) (Panel b) of medial forebrain bundle (MFB) in study subjects. RD_t_ is given in 10^−3^ cm^2^/s. A lower rating of pain intensity is associated with higher FA_t_ and lower RD_t_. This could be interpreted as that people with a higher degree of myelination of the MFB tract experience less pain

Rating of pain intensity correlated negatively with FA_t_ (Figure 3a) and positively with RD_t_ (Figure 3b). Correlation analysis of pain intensity rating and AD_t_, as well as pain intensity rating and FW was not significant (see Table 1).

**TABLE 1:** Results of correlation analyzes

	Pain intensity rating		
	*r*	*p*	*p*‐FDR corrected
MFB			
FA_t_	−.372	**.021**	**.043**
RD_t_	.383	**.018**	**.043**
AD_t_	.001	.994	.994
FW	−.120	.473	.630

*Note:* Pearson correlations coefficient (*r*) of brain measures, rating of pain intensity, and the corresponding *p*‐values (*p*) are listed. The *p*‐values of FA_t_, RD_t_, AD_t_, and FW were corrected using the FDR procedure. Bold values denote statistical significance at the p < 0.05 level.

Abbreviations: AD_t_, free‐water corrected axial diffusivity; FA_t_, free‐water corrected fractional anisotropy; FDR, false discovery rate; FW, free‐water; MFB, medial forebrain bundle; RD_t_, free‐water corrected radial diffusivity.

## 4 DISCUSSION AND CONCLUSIONS

We conducted the present dMRI study to gain knowledge about the neuronal substrates of interindividual variability of pain sensitivity. We analyzed the association between microstructural dMRI measures of the MFB and rating of pain intensity to applied heat stimuli in 38 healthy men. Our results indicate a higher FA_t_ and lower RD_t_, which might be explained by a better (stronger) myelination status in MFB, are associated with more efficient pain control after heat stimuli. Analyses of FW and AD_t_ in MFB revealed no association with pain.

The MFB is part of the descending pain system where modulation of pain occurs. We assessed the association of interindividual pain intensity rating after heat stimuli as representing a temporary effect, and the microstructure of the MFB as representing a stable structural status. We predicted lower FA_t_ and higher RD_t_ of the MFB in subjects with a higher ability to modulate pain. In line with our hypothesis, we report that interindividual acute pain intensity rating after heat stimulation correlates negatively with FA_t_ and positively with RD_t_. Since higher myelination is expected to increase FA_t_ and decrease RD_t_, but not AD_t_, our result could suggest that individuals with increased axonal myelination of MFB experience less pain. One explanation for the reduced pain intensity rating in subjects with increased axonal myelination of MFB is that MFB connectivity is associated with effective control of pain sensation after heat stimulation. However, as our results rely on correlation analyses in a cross‐sectional study, we cannot identify whether a better effective control of pain causes axonal myelination of MFB (training induced changes, see (Scholz, Klein, Behrens, & Johansen‐Berg, 2009; Sevel, Boissoneault, Alappattu, Bishop, & Robinson, 2019)) or whether an increased axonal myelination of MFB results in better effective control of pain (genetic component, see (Chiang et al., 2009; Young, Lariviere, & Belfer, 2012)). We found no association between interindividual pain intensity rating and FW, suggesting no effect of extracellular free water of MFB on pain sensitivity in young, healthy men. Our results are in line with a study by Stein et al. (2012) that reported that interindividual pain sensitivity was associated with lower FA of white matter tracts connecting the PAG with the rACC and DLPFC in healthy subjects (Stein et al., 2012). Our study extends the published study on two major issues. First, the published study focused on FA as the only outcome variable, whereas we have explored additional measures of the fiber microstructure like RD_t_ and AD_t_ to explore whether alteration in microstructure of the white matter tract is associated with individual pain sensitivity. Second, the published study has been restricted to the fiber connections of PAG with rACC and DLPFC, whereas our study also includes connections from other structures of the limbic forebrain including the OFC, NAc and amygdala, which are important parts of the descending pain system, but possibly distinct pathways. Indeed, it has been shown that self‐regulation and nociceptive input mediate pain perception via different brain systems (Woo, Roy, Buhle, & Wager, 2015). In an fMRI study, Woo et al. (2015) revealed that the neurologic pain signature (a pattern of fMRI activity across thalamus, insula, secondary somatosensory cortex, ACC, PAG, and other brain regions that are sensitive and specific to physical pain (Wager et al., 2013)) mediated the effects of noxious input (different heat stimulation intensities), whereas self‐regulation of pain was mediated through functional connections between the NAc and ventromedial prefrontal cortex, subcomponents of the MFB.

Although we did not assess chronic pain intensities, as we only investigated healthy participants, the connections of the brain regions of the mesolimbic pathway have been shown to be important in the development of chronic pain. For instance, a longitudinal study by Baliki et al. (2012) revealed that a greater functional connectivity of the NAc with mPFC predicts pain chronification. Additionally, they have shown that alterations in connections to NAc and mPFC, measured by FA, predetermine the transition from acute to chronic back pain (Mansour et al., 2013). Furthermore, Vachon‐Presseau et al. (2016) reported that anatomical characteristics of the dorsal mPFC, amygdala, NAc circuitry predict chronic back pain. In addition, Zhang et al. (2020) reconstructed nine brainstem fiber tracts that are potentially involved in modulation of chronic pain. Three out of these nine tracts also overlap partially with the MFB tract of our study. The three tracts of interest to our study are the MFT (that connects the ventral DC and prefrontal cortex), the dorsal longitudinal fasciculus (DLF that connects the PAG and hypothalamus), and the nigrostriatal tract (NST that connects the VTA and NAc). The authors also assessed the relationship between FA of the brainstem tracts and two pain severity levels—“pain right now,” and “worst pain in last month.” While no significant association between FA in the MFT and NST tracts and pain severity levels was reported, there was a significant negative correlation between both pain levels and FA in the DLF tract. Given that the DLF tract partially overlaps with the MFB tract, the findings of the Zhang et al., 2020 study and our study indicate correspondence between the descending pain control pathways that determine the strength of both chronic pain (pain severity) and acute pain (pain sensitivity). Studies of acute pain are further important, as individuals that are hypersensitive to acute pain are prone to experience increased musculoskeletal pain (Georgopoulos et al., 2019) and increased postoperative pain (Abrishami et al., 2011). Further, people with a reduced ability to modulate acute pain are at a higher risk to develop chronic pain (Edwards, 2005). Although we did not investigate the structure of MFB in patients with chronic pain, the association of interindividual pain sensitivity (measured by pain intensity rating) and alteration in FA_t_ and RD_t_ of the MFB highlight that the mesolimbic pathway and the connection between frontal areas of the brain with the brainstem play a major role in pain perception.

There are several limitations of the present study. First, our study focused on pain perception in men. Thus, our conclusion should not be generalized to women. Second, the MFB is a bidirectional structure comprised of ascending and descending fibers. Therefore, a separation of the influence of structural changes within the ascending versus descending system is not possible. However, a new predictive coding theory of pain processing suggests a single pain modulation system with reciprocity connection rather than separate top‐down and bottom‐up systems (Buchel, Geuter, Sprenger, & Eippert, 2014; Grahl, Onat, & Buchel, 2018; Wiech, 2016). Third, we did not delineate subcomponents of the MFB. However, we visualized the PAG region and a subset of fibers of the MFB in close relationship with PAG in Figure 2. We expect to investigate the segments of the MFB connecting exclusively to the PAG in future studies. Fourth, the data acquired for this study did not include a reverse phase‐encoding direction (AP and PA) acquisition as it was not commonly used at that time. Consequently, the present study did not include any EPI geometric distortion correction. However, we were able to consistently trace the MFB in all subjects (at least 32 streamlines per subject). Finally, we only analyzed healthy subjects. Consequently, our drawn assumptions on possible association of structural changes of the MFB and the risk to develop chronic pain should be investigated in further studies.

In summary, the results of the present study provide evidence that the microstructure of the MFB, possibly myelination, is associated with interindividual rating of pain intensity in healthy subjects. This highlights the influence of the mesolimbic pathway to the interindividual variability of pain sensitivity in acute pain and offers new research opportunities towards understanding the course of chronic pain (Jakobs, Fomenko, Lozano, & Kiening, 2019).

## Supporting information


**TABLE S1** Results of correlation analyzes

## Data Availability

The data of our study "Microstructural Alterations in Medial Forebrain Bundle are Associated with Pain Sensitivity" will be made available via a request to the authors.
