# Supplementary material for: Microstructural alterations in medial forebrain bundle are associated with interindividual pain sensitivity
Source: Hum Brain Mapp. 2020 Nov 10;42(4):1130–7. doi: 10.1002/hbm.25281 (PMC7856635; doi:10.1002/hbm.25281)
Supplement: Supplementary file 1 — TABLE S1 Results of correlation analyzes [file HBM-42-1130-s001.docx]

**Table S1. Results of correlation analyzes**

|  | **Pain intensity rating** | |
| --- | --- | --- |
|  | ***r*** | ***p*** |
| **Thalamo-occipital tract**  **Left hemisphere:**  FA_t_  RD_t_  AD_t_  FW  **Right hemisphere:**  FA_t_  RD_t_  AD_t_  FW | -0.055  0.063  0.056  0.064^§^  -0.201  0.249  0.140  -0.030 | 0.743  0.705  0.739  0.701^§^  0.226  0.132  0.401  0.860 |

Pearson correlations coefficient (*r*) of brain measures, rating of pain intensity and the corresponding *p*-values (*p*) are listed. § Spearman correlations coefficient and corresponding p-value due to non normally distributed data. FA_t_ = free-water corrected fractional anisotropy, RD_t_ = free-water corrected radial diffusivity, AD_t_ = free-water corrected axial diffusivity, FW = free-water.
